# Various Mechanisms Involve the Nuclear Factor (Erythroid-Derived 2)-Like (NRF2) to Achieve Cytoprotection in Long-Term Cisplatin-Treated Urothelial Carcinoma Cell Lines

**DOI:** 10.3390/ijms18081680

**Published:** 2017-08-02

**Authors:** Margaretha A. Skowron, Günter Niegisch, Philipp Albrecht, Gommert van Koeveringe, Andrea Romano, Peter Albers, Wolfgang A. Schulz, Michèle J. Hoffmann

**Affiliations:** 1 Department of Urology, Medical Faculty, Heinrich-Heine-University, Duesseldorf 40225, Germany; Margaretha.Skowron@hhu.de (M.A.S.); Guenter.Niegisch@hhu.de (G.N.); peter.albers@med.uni-duesseldorf.de (P.A.); Wolfgang.Schulz@hhu.de (W.A.S.); 2 Department of Neurology, Medical Faculty, Heinrich-Heine-University, Duesseldorf 40225, Germany; phil.albrecht@gmail.com; 3 Department of Urology, Maastricht University Medical Centre, Maastricht 6202AZ, The Netherlands; g.van.koeveringe@mumc.nl; 4 Department of Obstetrics and Gynaecology, GROW-School for Oncology & Developmental Biology, Maastricht University Medical Centre, Maastricht 6229HX, The Netherlands; a.romano@maastrichtuniversity.nl

**Keywords:** urothelial carcinoma (UC), cisplatin resistance, nuclear factor (erythroid-derived 2)-like 2 (NRF2), sequestosome 1 (p62/SQSTM1), Kelch-like ECH-associated protein 1 (KEAP1), cytoprotection, glutathione, nuclear factor kappa-light-chain-enhancer of activated B cells (NF-κB) pathway, hippo pathway

## Abstract

Therapeutic efficacy of cisplatin-based chemotherapy for advanced-stage urothelial carcinoma (UC) is limited by drug resistance. The nuclear factor (erythroid-derived 2)-like 2 (NRF2) pathway is a major regulator of cytoprotective responses. We investigated its involvement in cisplatin resistance in long-term cisplatin treated UC cell lines (LTTs). Expression of NRF2 pathway components and targets was evaluated by qRT-PCR and western blotting in LTT sublines from four different parental cells. NRF2 transcriptional activity was determined by reporter assays and total glutathione (GSH) was quantified enzymatically. Effects of siRNA-mediated NRF2 knockdown on chemosensitivity were analysed by viability assays, γH2AX immunofluorescence, and flow cytometry. Increased expression of NRF2, its positive regulator p62/SQSTM1, and elevated NRF2 activity was observed in 3/4 LTTs, which correlated with KEAP1 expression. Expression of cytoprotective enzymes and GSH concentration were upregulated in some LTTs. NRF2 knockdown resulted in downregulation of cytoprotective enzymes and resensitised 3/4 LTTs towards cisplatin as demonstrated by reduced IC_50_ values, increased γH2AX foci formation, and elevated number of apoptotic cells. In conclusion, while LTT lines displayed diversity in NRF2 activation, NRF2 signalling contributed to cisplatin resistance in LTT lines, albeit in diverse ways. Accordingly, inhibition of NRF2 can be used to resensitise UC cells to cisplatin, but responses in patients may likewise be variable.

## 1. Introduction

Bladder cancer is the 9th most common tumour world-wide and the most common cancer of the urinary tract [1]. About 90% of bladder cancers in industrialised countries are urothelial carcinomas (UC), which include muscle-invasive cancers, papillary tumours, and carcinoma in situ (CIS); the remaining 10% are mostly squamous cell carcinoma and adenocarcinoma [2,3]. Curative treatment by surgical approaches can be achieved in locally confined UC, but treatment of advanced stage or metastatic UC is generally palliative [4,5,6]. In these patients, cisplatin-based chemotherapy is the standard of care, whereas carboplatin-based combinations or monotherapies are less efficient [7,8]. Cisplatin-based regimens are also applied as neoadjuvant chemotherapy prior to cystectomy, but not all cancers respond and incomplete response is associated with poor prognosis [9]. Inherent or acquired resistance to cisplatin is therefore a critical factor in the treatment of UC.

Resistance to cisplatin can be due to various mechanisms [10,11], including activation of transcription factors promoting cell survival such as nuclear factor kappa-light-chain-enhancer of activated B cells (NF-κB) [12] or Yes-associated protein 1 (YAP1) [13]. In particular, many studies have implicated the nuclear factor (erythroid-derived 2)-like 2 (NRF2/*NFE2L2*), a regulator of cytoprotective enzymes, glutathione biosynthesis and metabolism that controls cellular responses towards oxidative stress [14,15,16,17].

NRF2 is a cap’n’collar basic-region leucine zipper transcription factor consisting of a large p45 and a small p18 subunit [18]. Under resting conditions it is polyubiquitinated by the Kelch-like ECH-associated protein 1 (KEAP1)-Cullin 3 (CUL3) E3 ligase complex which leads to its degradation. Under oxidative stress, KEAP1 instead is polyubiquitinated at Lys-63 and degraded. Accumulating free NRF2 may then translocate to the nucleus [17,19,20,21,22,23,24] where it heterodimerises with other transcription factors like small Maf proteins (MafF, MafG, or MafK) in order to bind to cis-regulatory antioxidant responsive elements (ARE) and to thereby activate transcription of genes controlling cellular responses to, e.g., oxidative stress [18,19,24,25,26,27]. The NRF2/KEAP1 interaction is further regulated by Sequestosome 1 (p62/SQSTM1), which competes with KEAP1 for NRF2 via its KEAP1 interacting region (KIR) motif to promote NRF2 release and translocation to the nucleus [28]. The p62/SQSTM1 promoter contains an ARE through which NRF2 promotes SQSTM1 transcription, thereby creating a positive feedback loop [28]. At several points, NRF2 signalling interfaces with NF-κB pathways in the control of downstream targets, for example by competition for the CREB-binding protein (CBP) [16].

Constitutive NRF2 activation is common in cancer [29] and facilitates resistance to cisplatin, as in cisplatin-resistant RT-112 bladder cancer cells and in murine squamous cell carcinoma models [19,30], by inducing cytoprotective enzymes, glutathione (GSH) biosynthesis and recycling, glutathione transferases, and MRP drug conjugate exporters [19,30,31,32,33]. In particular, the association between elevated cellular levels of GSH and cisplatin resistance suggests that conjugation with GSH can be an important step in cisplatin inactivation in MCF7, U87MG, U251MG, and U138MG cell lines [34,35]. The rate-limiting factors for GSH biosynthesis are the γ-glutamylcysteine ligase catalytic and modifier subunits GCLC and GCLM as well as the heterodimeric cysteine/glutamate exchange transporters SLC3A2 and SLC7A11 [21,36,37,38,39]. GSH content and glutathione S-transferase (GST) expression have been associated with tumour progression and cisplatin resistance in urothelial carcinoma T-24 cells and in urothelial tumour tissues [40,41].

As in some other cancer types, a significant fraction of UC contain mutations in the *NFE2L2* gene, typically missense mutations in the Neh2 domain required for KEAP1 interaction that are expected to cause NRF2 overexpression [42,43]. Likewise, as in other cancers, high NRF2 expression is associated with poor prognosis in UC. Experimentally, cisplatin resistance was linked to NRF2 activation in the UC cell line RT-112 which was likely caused by loss of KEAP1 [19]. Nevertheless, not many functional studies have been performed addressing the role of the NRF2 pathway in cisplatin resistance in a panel of several cell lines from one tumour entity or, with the specific interest of our study, in different urothelial carcinoma cell lines.

In order to study cisplatin resistance mechanisms in detail, we have previously established cisplatin-resistant sublines from four different UC cell lines (UCCs) by long-term treatment with escalating cisplatin doses over several months. Development of resistance was accompanied by significant phenotypic changes with increases in markers of epithelial-to-mesenchymal transition (EMT) and canonical WNT pathway target genes [44,45]. In the present study, we investigated NRF2 and its related pathways in these LTT cells.

## 2. Results

### 2.1. Increased Expression and Transcriptional Activity of Nuclear Factor Erythroid 2-Related Factor 2 (NRF2) in Long-Term Cisplatin Treated Cell Lines (LTTs)

**Figure 1:**
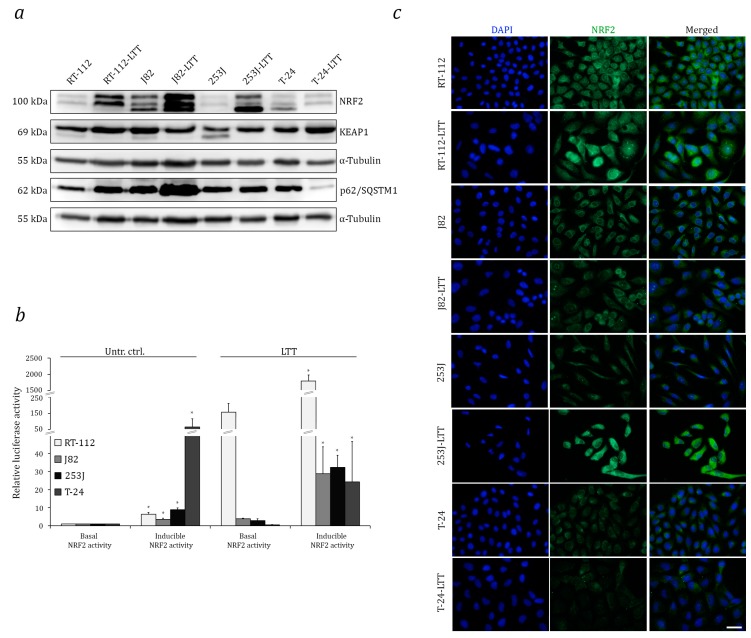
Increased expression and transcriptional activity of NRF2 in LTTs. (**a**) NRF2, KEAP1, and p62/SQSTM1 protein expression was measured in LTTs and their parental cell lines by Western blotting. As a loading control, α-Tubulin was stained. (**b**) Luciferase reporter activity was determined 72 h after transfection with pGL3-8xARE for basal NRF2 activity and in combination with NC16 pCDNA3.1 FLAG NRF2 for inducible NRF2 activity. Values represent the mean ± SD of biological triplicates. *p* < 0.05 * in basal vs. inducible NRF2 activity. (**c**) Immunofluorescence staining for NRF2 (green) in LTTs and their parental cell lines. DAPI staining (blue) was used to visualise nuclei. Scale bars = 50 μm.

We first evaluated expression of NRF2 and its regulators across a panel of UCCs covering the heterogeneity of the disease and then, in particular, compared the four long-term cisplatin treated LTTs with their parental cell lines. Across the UCCs, NRF2 and KEAP1 protein expression were heterogeneous and tended towards the expected inverse pattern, whereas p62/SQSTM1 expression was more uniform (Figure S1a). NRF2 protein levels were significantly increased in three out of four LTT cell lines, except in T-24-LTT, compared to the parental cell lines (Figure 1a). KEAP1 protein was only diminished in J82-LTT, which also overexpressed p62/SQSTM1. In T-24-LTT, conversely, KEAP1 was increased and p62/SQSTM1 was diminished (Figure 1a). Elevation of NRF2 protein in the three LTTs was constitutive and not due to induction by culturing with cisplatin, as shown by comparing cisplatin treated LTTs and LTTs without treatment for 10 days with their parental cell lines with or without cisplatin treatment (Figure S1b).

NRF2 and KEAP1 mRNA expression were both significantly decreased in three LTTs compared to their parental cell lines, whereas p62/SQSTM1 mRNA was upregulated. T-24-LTT was again the exception with no significant difference in NRF2 or p62/SQSTM1 mRNA, but increased KEAP1 mRNA expression (Figure S1c). 

NRF2 transcriptional activity was measured by a reporter assay following transfection with an ARE-luciferase expression construct. Compared to the untreated control, ARE-dependent luciferase activity was increased in RT-112-LTT, J82-LTT, and 253J-LTT, but was significantly decreased in T-24-LTT. Co-transfection with an NRF2 expression construct significantly increased luciferase activity in all four LTTs as well as in the parental lines; however, in T-24-LTT the inducible NRF2 luciferase activity remained lower compared to the parental cell line (Figure 1b). Remarkably, basal and inducible luciferase activity in RT-112-LTT was 100- and 1000-fold increased, respectively, in comparison to their parental cell line. These differences among LTT lines could relate to the differences in the cisplatin end concentrations they tolerated, as described in the Material and Methods. Immunofluorescence staining for NRF2 revealed increased expression in RT-112-LTT and 253J-LTT and nuclear staining in three out of four LTTs, with T-24-LTT being the only exception (Figure 1c).

As a possible cause of enhanced NRF2 expression and transcriptional activity, we searched for mutations in the *NFE2L2* and *KEAP1* genes. According to the TCGA data on UC [46,47], missense mutations in *NRF2/NFE2L2* are usually located in exon 2 and were detected in 14 (11%) of 126 sequenced UC. *KEAP1* was altered in 10 (7%) of 126 sequenced cases/patients, with 4 missense and 1 truncating mutation (Figure S2a). Deletion of *NFE2L2* exon 2 represents an alternative mechanism for activation of *NRF2* in a subset of squamous lung and head-and-neck cancers [48]. Somatic mutations of the *KEAP1* gene, especially in the BTB domain encoded by exon 2 have been identified in several solid cancers, e.g., NSCLC and gastric adenocarcinoma [49,50]. Exons 2 of *NFE2L2* and *KEAP1* were detectable in all four UCCs and their LTT sublines (Figure S2b). Sanger sequencing revealed no changes in *NFE2L2* exon 2 (data not shown), but a non-silent missense mutation in *KEAP1* exon 2, namely c. 334A>T (T112S), in RT-112-LTT, which was not present in the parental cell line (Figure S2c). Interestingly, an independently obtained RT-112 cisplatin-resistant cell line, RT-112-cp [51] contained the same mutation (Figure S2d,e). Despite introducing a serine residue this previously unreported mutation is not predicted to change phosphorylation by in silico analysis [52], but according to the KEAP1 crystal structure (data not shown, PDB ID 5NLB [53,54]), the amino acid change could lead to a conformational change impairing KEAP1 binding to Cullin-3 and thereby interfere with NRF2 degradation. It remains to be elucidated why this mutation occurs preferentially during treatment of RT-112. Two additional cisplatin-resistant cell lines derived by pulse-treatment, RT-112-R and J82-R [45] contained no alternations in exons 2 of *NFE2L2* and *KEAP1* (Figure S2d).

### 2.2. Induction of Cytoprotective Enzymes and Elevated Glutathione (GSH) Levels in LTTs

**Figure 2:**
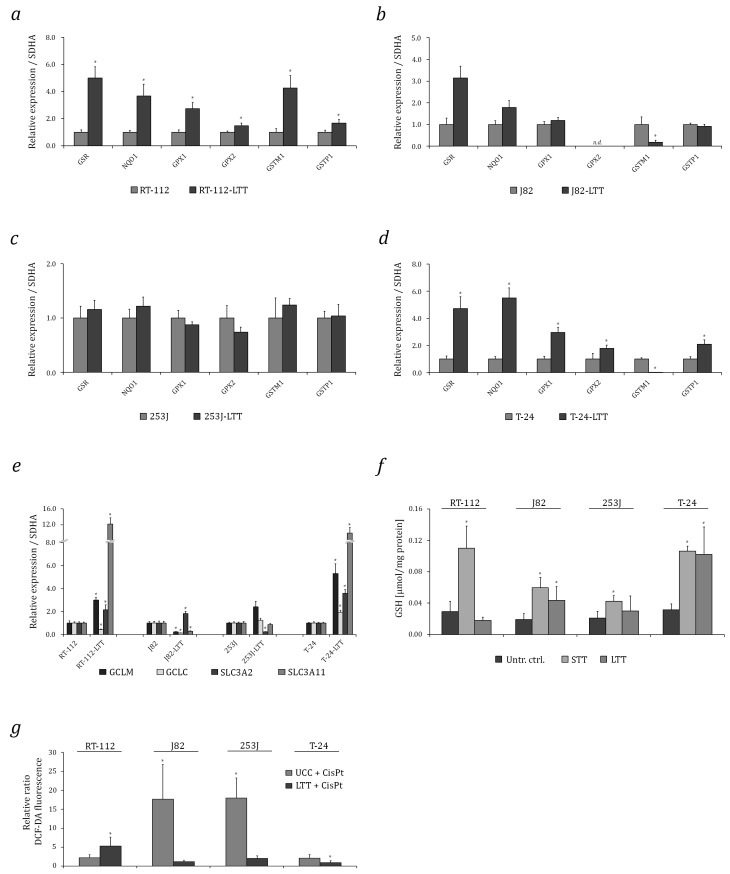
Induction of cytoprotective enzymes and GSH elevation in LTTs. GSR, NQO1, GPX1, GPX2, GSTM1, and GSTP1 mRNA expression in (**a**) RT-112-LTT, (**b**) J82-LTT, (**c**) 253J-LTT, (**d**) T-24-LTT and their parental cell lines was measured by qRT-PCR. (**e**) GCLC, GCLM, SLC3A2, and SCL7A11 mRNA expression in LTTs and their parental cell lines were measured by qRT-PCR. Expression levels in the untreated control were set as 1. SDHA mRNA was used as a reference and relative expression was calculated by the 2^−ΔΔ*C*t^ method. (**f**) GSH content normalised to total protein measured after 72 h cisplatin treatment in parental UCCs and LTTs compared to untreated controls. (**g**) Relative ratio of intracellular ROS accumulation as determined by DCFH-DA fluorescence staining and flow cytometry in parental UCCs and LTTs after 72 h cisplatin treatment depicted as a bar graph. Values represent the mean ± SD of biological quadruplicates, * *p* < 0.05.

Expression of NRF2 target genes was quantified by qRT-PCR (Figure 2a–d, Table S1). Significantly increased expression of the cytoprotective enzyme genes GSR, NQO1, GPX1, GPX2, GSTM1, and GSTP1 was observed in RT-112-LTT compared to their parental cells (Figure 2a) and, except for GSTM1, also in T-24-LTT (Figure 2d). J82-LTT expressed higher GSR mRNA compared to its parental cell line (Figure 2b). Cytoprotective enzymes mRNAs remained mostly unchanged in 253J-LTT compared to its parental cell line (Figure 2c).

Among GSH biosynthetic genes, GCLM mRNA was elevated in three out of four LTTs, albeit not in J82-LTT, whereas GCLC mRNA expression was generally unchanged (Figure 2e). The mRNA of the SLC3A2 transporter was significantly elevated in RT-112-LTT, J82-LTT, and T-24-LTT compared to their parental cell lines. SLC7A11 mRNA expression was significantly increased in RT-112-LTT and T-24-LTT, but remained unchanged or were even significantly decreased in 253J-LTT and J82-LTT, respectively, compared to their parental cell lines (Figure 2e, Table S1).

Total GSH was significantly increased in J82-LTT and T-24-LTT, but not in RT-112-LTT and 253J-LTT (Figure 2f). Interestingly, total GSH increased significantly after short-term cisplatin treatment (STT) of parental cell lines (Figure 2e), without changes in the NRF2 protein level (compare Figure S1b). Moreover, compared to their parental cell lines, significantly lower intracellular ROS concentrations were detected after cisplatin treatment in J82-LTT, 253J-LTT and T-24-LTT, but not in RT-112-LTT, where significantly higher levels were observed after cisplatin treatment (Figure 2g and Figure S3).

### 2.3. NRF2 as A Target to Sensitise Cisplatin-Resistant Urothelial Carcinoma Cell Lines (UCCs)

**Figure 3:**
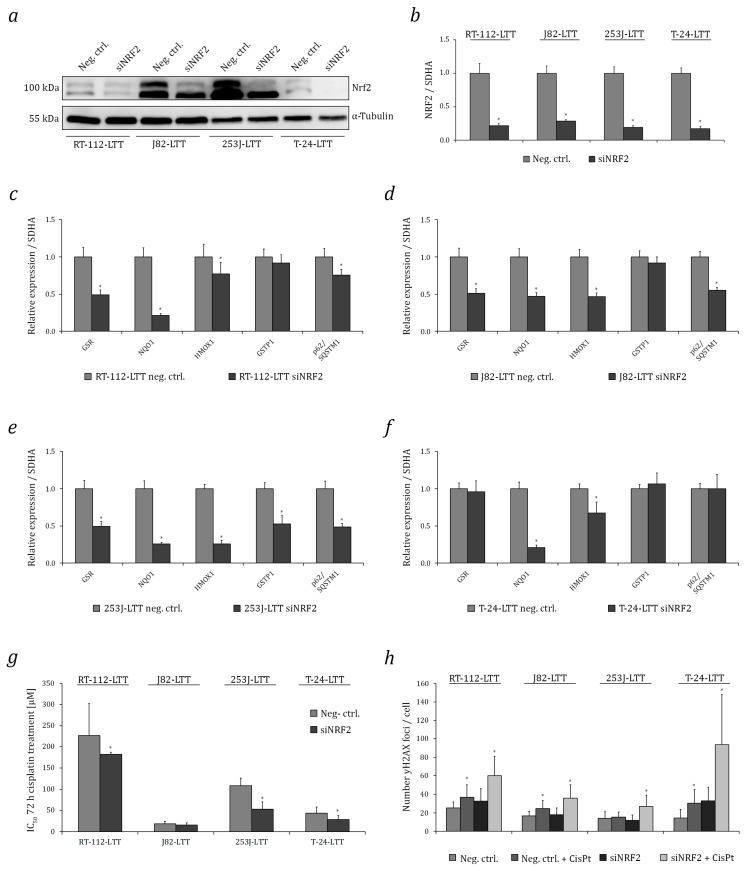
NRF2 knockdown sensitises LTTs towards cisplatin by increasing DNA damage. NRF2 protein (**a**) and mRNA (**b**) expression was measured in LTTs after siNRF2-mediated knockdown and their negative controls. As a loading control, α-Tubulin was detected. GSR, NQO1, HMOX1, GPX1, GSTP1, and p62/SQSTM1 mRNA expression in siNRF2-transfected (**c**) RT-112-LTT, (**d**) J82-LTT, (**e**) 253J-LTT, (**f**) T-24-LTT and their negative controls were measured by qRT-PCR. Expression levels in the negative control were set as 1. SDHA mRNA was used as a reference and relative expression was calculated by the 2^−ΔΔ*C*t^ method. (**g**) Cell viability was measured 72 h after cisplatin treatment by CellTiterGlo assay in LTTs transfected with siRNA targeting NRF2 or a non-targeting negative control siRNA. (**h**) Quantification of pH2A.X Ser139 foci by immunofluorescent staining in LTTs transfected with siRNA targeting NRF2 or a non-targeting negative control siRNA and treated or not treated with cisplatin after 72 h. Values represent the mean ± SD of biological duplicates, * *p* < 0.05.

**Figure 4:**
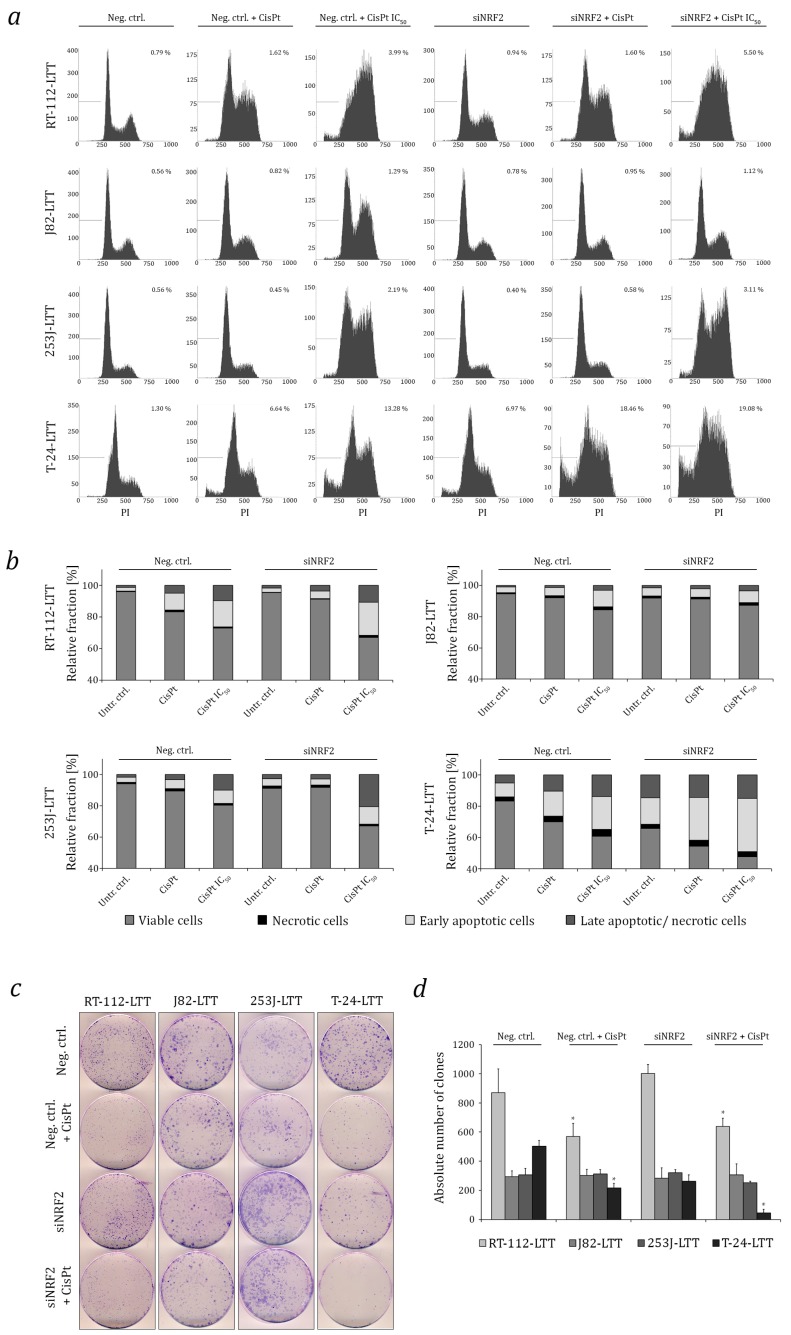
NRF2 knockdown sensitises LTTs towards cisplatin by induction of cell death. (**a**) Changes in cell-cycle distribution and amount of dead cells (as sub-G1 fraction) in siNRF2-transfected LTTs 72 h after cisplatin treatment (resistance and IC_50_ concentrations) were measured by flow cytometry. Treatment with non-targeting siRNA served as a negative control. (**b**) Induction of necrosis and apoptosis was analysed in siNRF2- or control siRNA-transfected LTTs after 72 h cisplatin treatment (resistance and IC_50_ concentrations) by combined Annexin V and PI staining with subsequent flow cytometry as shown in a bar graph. (**c**) Colony formation assay of siNRF2- or control siRNA-transfected LTTs treated with resistance concentrations of cisplatin for 72 h. Cell clones were stained by Giemsa. (**d**) Quantification of colonies shown in (**c**). Values represent the mean ± SD of biological duplicates and technical triplicates, * *p* < 0.05.

Efficient knockdown of NRF2 mRNA and protein was achieved in all four LTTs (Figure 3a,b). Consequently, compared to treatment with a non-targeting control siRNA, mRNA expression of the NRF2 targets NQO1, GSR, HMOX1, GSTP1, GPX1, and p62/SQSTM1 was downregulated in almost all four cell lines (Figure 3c–f). Cisplatin sensitivity increased, with statistically significant changes in the IC_50_ values for cisplatin in RT-112-LTT, 253J-LTT, and T-24-LTT, albeit not in J82-LTT (Figure 3g). Concomitantly, following cisplatin treatment, increased γH2AX formation indicative of increased DNA damage was observed by immunocytochemistry in all four siNRF2-treated LTTs compared to LTTs transfected with negative control siRNA. Again, the difference was statistically significant in RT-112-LTT, 253J-LTT, and T-24-LTT, but not in J82-LTT (Figure 3h and Figure S4). Cell cycle analysis as well as Annexin V staining and subsequent analysis by flow cytometry revealed increased sub-G1 fractions and a higher number of apoptotic cells following treatment with cisplatin in three out of four siNRF2 treated LTTs, except for J82-LTT (Figure 4a,b and Figure S5). Notably, following NRF2 knockdown in T-24-LTT, apoptosis was increased and clonogenicity was diminished even in the absence of cisplatin treatment (Figure 4c,d).

### 2.4. Relation of Increased NRF2 Expression to Hippo and Nuclear Factor Kappa B (NF-κB) Pathways in LTTs

**Figure 5:**
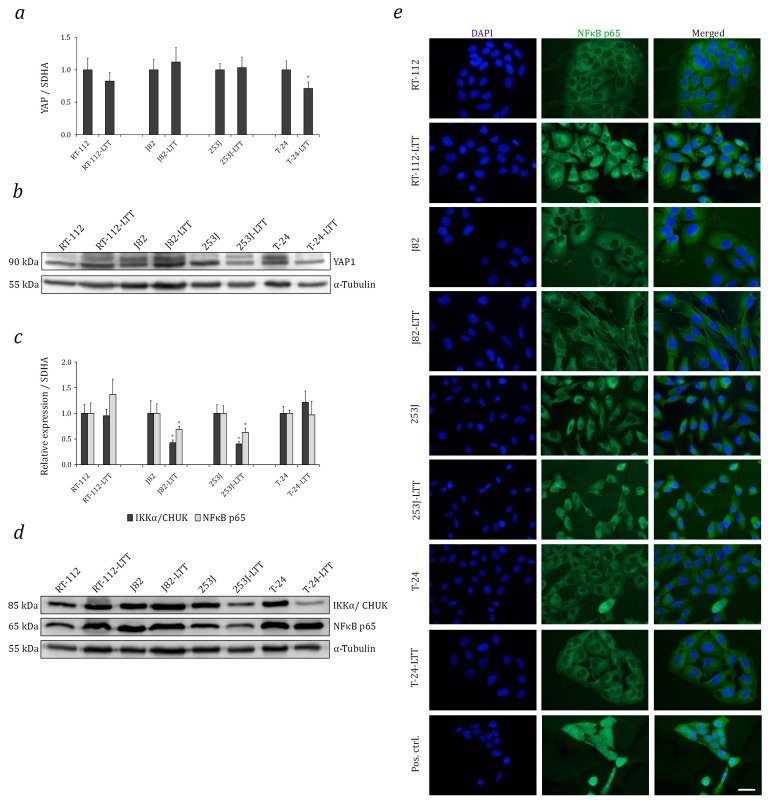
Increased NRF2 expression is independent of Hippo or NF-κB pathway changes in LTTs. (**a**) YAP1 mRNA expression in LTTs and their parental cell lines was measured by qRT-PCR. Expression levels in the untreated control were set as 1. SDHA mRNA was used as a reference and relative expression was calculated by the 2^−ΔΔ*C*t^ method. Values represent the mean ± SD of biological triplicates. (**b**) YAP1 protein expression was measured in LTTs and their parental cell lines. As a loading control, α-Tubulin was detected. (**c**) IKKα and NF-κB p65 mRNA expression in LTTs and their parental cell lines were measured by qRT-PCR. (**d**) IKKα and NF-κB p65 protein expression was measured in LTTs and their parental cell lines. (**e**) Immunofluorescence stainings for NF-κB p65 (green) in LTTs and their parental cell lines. DAPI staining (blue) was used to visualise nuclei. Scale bars = 50 μm, * *p* < 0.05.

To study crosstalk between the NRF2, Hippo, and NF-κB pathways, we first assessed YAP1, IKKα/*CHUK*, and NF-κB p65/*RELA* protein expression across an UC cell line panel. With the exception of 5637, IKKα and NF-κB p65 protein expression were very similar among the tested UCCs, whereas YAP1 protein expression was heterogeneous. Notably, J82 and T-24 were among the cell lines with high expression (Figure S1a). In most LTTs, YAP1 mRNA and protein expression were unchanged compared to the parental cell lines, except for a significant decrease in T-24-LTT (Figure 5a,b, Table S1). Further, IKKα and NF-κB p65 mRNA expression was significantly decreased in J82-LTT and 253J-LTT compared to their parental cell lines (Figure 5c, Table S1). IKKα and NF-κB p65 mRNA expression were accordingly decreased in 253J-LTT. However, both IKKα and NF-κB p65 proteins appeared unchanged in J82-LTT (Figure 5d). By immunofluorescence staining, NF-κB p65 was exclusively cytoplasmically localised in all four UCCs and their LTT sublines (Figure 5e).

## 3. Discussion

This study revealed NRF2 as an important factor in each of four independent long-term cisplatin treated (LTT) urothelial carcinoma cell lines (UCCs), fitting the established notion of NRF2 as a generally important factor in the development of chemoresistance and as a rational target to restore chemosensitivity [39]. Upon closer analysis, however, the cell lines differed in the extent of NRF2 expression and activation, its upstream regulation, and the expression of its downstream targets. Thus, our study of four different cell lines from the same tumour entity highlights the diversity in the modes of NRF2 activation and the ensuing gene expression changes. We consider it likely that a similar degree of diversity in NRF2 activation will occur in patients during cancer treatment with cisplatin-containing regimens, especially in cancer types with pronounced heterogeneity like UC. Therefore, while our data, on the one hand, support the notion of NRF2 as an important factor in chemoresistance, they suggest, on the other hand, that targeting NRF2 may not lead to a uniform response and NRF2 activation needs to be considered in a broader perspective, also including interfering and crosstalk pathways.

In detail, the three resistant cell lines RT-112-LTT, J82-LTT, and 253J-LTT expressed more NRF2 protein, which was localised in the nucleus, and was associated with basal and inducible NRF2 activity in reporter assays. NRF2 protein expression was unchanged in a fourth resistant cell line, T-24-LTT, compared to parental T-24 cells. SiRNA-mediated knockdown of NRF2 decreased IC_50_ values for cisplatin in RT-112-LTT, 253J-LTT, and T-24-LTT and this sensitisation was paralleled by increased γH2AX foci formation, in line with the presumed functions of NRF2. Unexpectedly, only a minor sensitisation was achieved in J82-LTT by NRF2 knockdown, which, also unexpectedly, instead induced apoptosis in T-24-LTT even in the absence of treatment (see detailed discussion below). Concordantly with our results, NRF2 knockdown sensitised the lung carcinoma cell line A549 to cisplatin, whereas its stable overexpression enhanced cisplatin resistance of breast adenocarcinoma and neuroblastoma cells MDA-MB-231 and SH-SY5Y [55]. Similarly, inhibition of KEAP1 stabilised NRF2 expression in SCC stem cells and rendered them resistant to cisplatin [30].

As a possible cause of increased NRF2 expression, we found elevated p62/SQSTM1 protein and mRNA expression in RT-112-LTT, J82-LTT, and 253J-LTT. Analogously, cisplatin-resistant ovarian cancer SKOV3 cells expressed more p62/SQSTM1 and were resensitised upon knockdown of p62/SQSTM1 [56,57]. The deletion of *NFE2L2* exon 2 represents an alternative mechanism for activation of NRF2 in squamous carcinomas [48]. In hepatocarcinogenesis most NRF2 mutations are located in the regions coding for the DLG or ETGE motives, which bind to the Kelch domain in KEAP1 [58,59]. However, exon 2 was present and unchanged in all four UC LTT. Instead, we detected a mutation near the BTB domain of KEAP1, c. 334A>T (T112S), in RT-112-LTT, which, despite the newly introduced serine, likely does not affect protein phosphorylation. Nevertheless, the T112 side chain interacts with the neighbouring amino acid backbone to stabilise a protein loop, and its replacement by a serine could impair interaction with Cullin 3 [60]. In their cisplatin-resistant RT-112 subline, Hayden et al. [19] observed loss of KEAP1 expression, but its cause was not elucidated. In other cancer types, KEAP1 mutations in the first Kelch domain (e.g., G333C in A549 cells) and in the intervention region (IVR, D236H in H460 cells) modified NRF2 signalling and influenced platinum sensitivity [50]. Of note, in addition to p62/SQSTM1, other negative regulators of the NRF2–KEAP interaction, such as Gankyrin (PSMD10, 26S proteasome non-ATPase regulatory subunit 10) [61,62] could be involved and may deserve further investigation. In theory, KEAP1-mediated control of NRF2 might also be disrupted by the cyclin inhibitor p21^Cip1/Waf1^, which competes with oxidised KEAP1 for binding to the NRF2 DLG motif to enhance the stability of the transcription factor [21,63,64,65,66]. In the current study, p21 was not differentially expressed between parental and LTT cell lines (data not shown) and thus appeared unrelated to NRF2 expression and activity in LTTs. Moreover, some UCCs, including RT-112, do not express functional p21 because of frameshift mutations.

Many cytoprotective enzymes inducible by NRF2 [67] were upregulated in RT-112-LTT compared to its parental cell line, similar to the observations by Hayden et al. in their independently derived cisplatin-resistant RT-112 line [19]. Similar results were obtained for T-24-LTT even though these cells did not display increased NRF2 protein expression. In J82-LTT and 253J-LTT most NRF2 targets were not significantly induced. In most LTTs, though, NRF2 knockdown diminished mRNA expression of downstream cytoprotective enzymes, such as GSR, NQO1, HMOX1, and GSTP1, as well as the NRF2 target p62/SQSTM1.

Increased glutathione availability with consequently decreased intracellular ROS formation is another potential consequence of NRF2 activation that protects against cisplatin by forming extrudable conjugates and by ameliorating oxidative stress. For instance, higher GSH levels were observed in cisplatin-resistant A2780 ovarian carcinoma cells and the U373MG glioblastoma cell line [68,69]. Another cisplatin-resistant ovarian carcinoma cell line containing high levels of GSH could be sensitised by NRF2 inhibition [70]. In our study, elevated GSH content and decreased intracellular ROS production was found in J82-LTT and especially in 253J-LTT and T-24-LTT, compared to their respective parental lines. Elevated GSH corresponded with decreased oxidative stress following cisplatin treatment. Surprisingly, total GSH levels remained unchanged and intracellular oxidative stress was increased in RT-112-LTT compared to its parental cell line. Conceivably, GSH is efficiently conjugated with cisplatin in RT-112-LTT and exported by the overexpressed multidrug resistance-associated protein 2 (MRP2), resulting in a decreased steady-state GSH content. Key determinants of glutathione biosynthetic capacity are the cystine–glutamate transporters SLC7A11 and SLC3A2, both of which have previously been associated with cisplatin resistance [37,38]. These were upregulated in RT-112-LTT and T-24-LTT. Specifically, SLC7A11 overexpression as a consequence of diminished expression of its negative regulator miRNA-27a has been identified as a mechanism of cisplatin resistance in derivatives of the EJ UC cell line [71]. In that study, the combination of low miRNA-27a and high SLC7A11 was also observed in cancer tissues, but in a relatively low fraction of cases. This finding thus further illustrates the diversity of mechanisms involved in NRF2 action in UC, in cell lines as well as in tumours.

The diversity of NRF2 regulation is further illustrated by the J82-LTT line, which presented high basal NRF2 protein expression and enhanced reporter gene activity. Nevertheless, efficient siRNA-mediated knockdown of NRF2 had minimal effects on cisplatin sensitivity in this resistant subline. Indeed, expression of cytoprotective enzymes and of genes involved in GSH transport and biosynthesis was largely unchanged in J82-LTT compared to its parental line. As nuclear immunofluorescence staining of NRF2 was not as prominent in J82-LTT as in RT-112-LTT and 253J-LTT, we suspect that NRF2 might be prevented from activating its target genes in a KEAP1 independent manner. An established mechanism that might explain this constellation involves phosphorylation of nuclear NRF2 by GSK3β and recognition of phosphorylated NRF2 by β-TrCP in the ubiquitin E3 ligase complex SCF (Skp1-Cullin-F-box), leading to its poly-ubiquitination and degradation [72,73,74,75]. Alternatively, negative regulators of the NRF2-MafK interaction like BACH1 [18] might account for the phenotype of this cell line.

Whereas NRF2 protein was increased in all other LTTs, it was unchanged in T-24-LTT compared to its parental cell line. Basal NRF2 activity was decreased, but luciferase activity was inducible, albeit to a lesser extent than in its parental cell line. Expression of p62/SQSTM1 remained essentially unchanged, but KEAP1 protein and mRNA were upregulated. However, the low amount of NRF2 present proved essential in this cell line, as siNRF2 knockdown not only sensitised T-24-LTT to cisplatin, but also induced apoptosis and decreased clonogenicity in the absence of cisplatin especially in this cell line. Suppression of cell proliferation by complete siNRF2-mediated knockdown has also been observed in the cholangiocarcinoma cell lines KKU-156 and KKU-100 and was similarly enhanced by cisplatin treatment [76]. The sensitivity of T-24-LTT to NRF2 knockdown may of course relate to the downregulation of cytoprotective and GSH biosynthetic enzymes by the treatment. Strikingly, many of these factors were elevated in T-24-LTT compared to the parental cell line, even though NRF2 expression and activity were not increased. This observation suggests that NRF2 cooperates with another pathway acting on its target genes in this cell line. A good candidate is the aryl hydrocarbon (AhR) pathway, which exhibits crosstalk with NRF2 signalling at several levels [16]. AhR is kept inactive in the cytoplasm by binding to a complex of Hsp90, XAP2, and p23 protein. Upon activation, AhR translocates into the nucleus where it heterodimerises with the aryl hydrocarbon receptor nuclear translocator (ARNT) at xenobiotic response elements (XREs) to activate transcription [75,77,78,79]. Many NRF2 targets also contain XREs [80,81].

Two other potentially interacting pathways, the Hippo and NF-κB pathways [11,12], were not altered in the LTTs. The major crosstalk between NRF2 and NF-κB pathways occurs through competition between the NF-κB p65 subunit and NRF2 for their common co-activator CBP. Following its activation by phosphorylation at Ser276, p65 suppresses transcription of ARE-dependent genes by depriving NRF2 of CBP [21,25,72]. Another factor mediating this crosstalk is p62/SQSTM1, which binds tumour necrosis factor (TNF) receptor associated factor 6 (TRAF6) via its TRAF6 binding domain (TB). Activation of TRAF6 leads to the phosphorylation of IKKβ, which phosphorylates IκB resulting in its ubiquitination and thereby the release of the NF-κB dimer consisting of p50 and p65 (Rel-A), which can then enter the nucleus for transcriptional regulation [26,28,82,83]. Moreover, the emerging role of the Hippo pathway has been described in cisplatin-resistant UC patient-derived xenograft models. There, YAP1 knockdown in T-24 cells increased sensitivity towards cisplatin by increasing DNA damage accumulation leading to apoptosis [13]. In our study, we did not observe a correlation between increased NRF2 activity and YAP1 protein expression.

In conclusion, our results indicate that NRF2—in different ways and to different extents—is a key player in the development of cisplatin resistance in UCCs and may constitute a reasonable target to combat chemoresistance in UC. Nevertheless, the diversity in NRF2 activation among the four tested LTTs highlights the complexity of cisplatin resistance even within one tumour entity and predicts that responses to NRF2 inhibition may likewise be highly variable in patients.

## 4. Materials and Methods

### 4.1. Cell Culture, Treatment, Plasmid Transfection, and siRNA-Mediated Knockdown

The human UC cell lines RT-112, VM-CUB-1, UM-UC-3, T-24, 639V, 253J, 5637, SW-1710, HT-1376, BFTC-905, and J82, kindly provided by M. A. Knowles (Leeds, UK), J. Fogh (New York, NY, USA), B. Grossmann (Houston, TX, USA), or the DSMZ (Braunschweig, Germany), were grown in DMEM GlutaMAX-I (Gibco, Darmstadt, Germany) containing 10% FCS. All cell lines were recently authenticated by standard DNA fingerprint analysis. IC_50_ doses were determined after treatment with a single IC_50_ dose of cis-diamminedichloroplatinum-II (cisplatin; Accord Healthcare, London, UK) for 72 h by MTT assay. For short-term experiments (STT) a single IC_50_ dose cisplatin was added for 72 h. As previously reported, long-term treated (LTT) UCCs were generated by adding cisplatin after every passage at escalating doses over months with constant end concentrations of 50, 3.3, 6.6, and 23 µM in RT-112-LTT, J82-LTT, 253J-LTT, and T-24-LTT, respectively [44]. Reporter plasmids NC16 pCDNA3.1 FLAG NRF2, a gift from Randall Moon (Addgene plasmid #36971, Cambridge, MA, USA) [84], and GL3–8xARE, kindly provided by R. Wolf (Dundee, UK) [85], were transfected using X-tremeGENE9 DNA Transfection Reagent (Roche, Basel, Switzerland) according to the manufacturer’s instructions. For siRNA-mediated knockdown, UCCs and LTTs were transfected with 10 nmol/L siNRF2 or a non-targeting control (#L-003755–00 and #D-001810–10-05, both Dharmacon, Lafayette, CO, USA) using Lipofectamine RNAiMAX Reagent (Thermo Fisher, Waltham, MA, USA) according to the manufacturer’s protocol. For colony formation assay cells were fixed in methanol before staining with Giemsa. Quantification of colonies was performed with ImageJ software 1.51k (National Institute of Mental Health, Bethesda, MD, USA) and a cell counter plugin.

### 4.2. Molecular Analyses

RNA isolation, cDNA synthesis, and quantitative real-time PCR were performed as previously described [44]. qRT-PCR was conducted using self-designed primers on the Lightcycler 96 system (Roche, Basel, Switzerland) (Table S2). For normalisation the housekeeping gene *SDHA* was used. DNA was extracted using the Blood & Cell Culture DNA Midi Kit according to the manufacturer’s protocol (Qiagen, Hilden, Germany).

### 4.3. Mutation Analysis by Sequencing

For *NFE2L2* and *KEAP1* mutation analysis 468 and 842 bp amplicons containing exon 2, respectively, were amplified following PCR from genomic DNA. Primer sequences are detailed in Table S3. PCR products were purified using the DNA Clean and Concentrator Kit (Zymo Research, Irvine, CA, USA) and analysed by Sanger sequencing. The interaction between the BTB domain of KEAP1 and Cullin 3 (PDB ID 5NLB [53,54]) was analysed by using PyMOL software version 1.5.0.4 (Schrödinger, Portland, OR, USA). The Protein Kinase Identification Server (PKIS) was used to predict changes in phosphorylation at the mutated site [52].

### 4.4. Measurement of Cell Viability

Cell viability was measured in quadruplicates by means of MTT assay (Sigma-Aldrich, St. Louis, MO, USA) and CellTiter-Glo assay (Promega, Fitchburg, WI, USA).

### 4.5. Flow Cytometry

Cell-cycle analyses were performed by staining the attached and supernatant cells with buffer containing 50 µg/mL propidium iodide (PI), 0.1% sodium citrate, and 0.1% Triton X-100 [86]. For assessing apoptotic cell death, attached and supernatant cells were incubated with 5 µL Annexin V-FITC (#31490013, Immunotools, Friesoythe, Germany) and 30 µg/mL PI in buffer containing 10 mM HEPES, 150 mM NaCl, 5 mM KCl, 5 mM MgCl_2_, and 1.6 mM CaCl_2_. For the quantification of intracellular ROS-concentration, cultured cells were incubated in medium without phenol-red and FCS containing 10 µM 2′,7′-dichlorodihydrofluorescein diacetate (DCFH-DA #4091-99-0, Santa Cruz Biotechnology, Dallas, TX, USA) for 30 min in the dark at 37 °C. Following washing with PBS, scratched cells were taken up in PBS. Flow cytometry analyses were performed using the Miltenyi MACSQuant Analyser with MACSQuant analysis software 2.6 (Miltenyi Biotec, Bergisch Gladbach, Germany).

### 4.6. Immunofluorescence

For staining of NF-κB p65 (#610868, BD Bioscience, San Jose, CA, USA, 1:100) and pH2A.X Ser139 (#2577, Cell signalling, Cambridge, UK, 1:100), cells were fixed on coverslips with 4% formaldehyde for 10 min and permeabilised at room temperature for 10 min in 0.5% Triton-X100 in PBS. For NRF2 staining (ab62352, Abcam, Cambridge, UK, 1:50), cells were fixed with methanol at −20 °C for 10 min and permeabilised at room-temperature for 10 min in 0.5% Saponin in PBS. Cells were blocked for one hour at room temperature in 1% BSA for NF-κB p65 and in 10% goat serum for NRF2 staining or 0.3 M glycine, and 0.1% Triton-X for pH2A.X Ser139 staining. Secondary antibodies Alexa Fluor 488 Goat Anti-Mouse IgG or Alexa Fluor 488 Goat Anti-Rabbit IgG (1:500, Life Technologies, Carlsbad, CA, USA) were added for 1 h in the dark at room-temperature. Cover slips were counter-stained with 0.5 mg/mL DAPI. Quantification of pH2A.X Ser139 foci was performed using the Focinator software tool [87].

### 4.7. Western Blot

Total protein was extracted by lysis for 30 min on ice in RIPA buffer pH 7.6 (150 mM NaCL, 1% Nonidet P-40, 0.5% sodium deoxycholate, 1% Triton X-100, 0.1% SDS, 1 mM EDTA, 50 mM Tris pH 7.6, 1% protease inhibitor cocktail (#P8340) and 1% phosphatase inhibitor (#P0044, both Sigma-Aldrich). Determination of protein concentrations and western blot analysis of whole-cell extracts was performed as described previously [88]. Primary antibodies were used against NRF2 (1:1000, ab62352; Abcam, Cambridge, UK), KEAP1 (sc-365626), p62/SQSTM1 (sc-28359), YAP (sc-398182; all 1:1000; Santa Cruz Biotechnology, Heidelberg, Germany), NF-κB p65 (#610868) and IKKα (#556532; both BD Bioscience, San Jose, CA, USA, 1:1000). Secondary antibodies were HRP-conjugated goat-anti-mouse or goat-anti-rabbit antibodies (1:5000, sc-2005 or sc-2004, Santa Cruz Biotechnology, Heidelberg, Germany). Anti-α-Tubulin (1:10000, B-512, Sigma-Aldrich) was used as loading control. Expression levels were visualised by WesternBright Quantum Kit (Biozym, Hessisch Oldendorf, Germany) or Super Signal West Femto (Thermo Fisher Scientific, Waltham, MA, USA) on the Li-COR C-DiGit Blot Scanner using Image Studio Digits 4.0 software (LI-COR Biosciences, Lincoln, NE, USA).

### 4.8. GSH Assay

Total glutathione was measured enzymatically by the method of Tietze [89,90,91] and normalised to cellular protein measured by the bicinchoninic acid-based method (Pierce, Thermo Fisher Scientific).

### 4.9. Use of the cBioPortal Data Base

NRF2/NFE2L2 and KEAP1 mutations in 413 bladder urothelial carcinoma samples (TCGA) were analysed using the cBioPortal for Cancer Genomics [46,47].

## Supplementary Materials

Supplementary materials can be found at www.mdpi.com/1422-0067/18/8/1680/s1.

## Abbreviations

LTT	Long-term cisplatin treated
LTTs	Long-term cisplatin treated cell lines
STT	Short-term cisplatin treated
UC	Urothelial carcinoma
UCCs	Urothelial carcinoma cell lines
SDHA	Succinate dehydrogenase complex
NRF2/NFEL2L	Nuclear factor (erythroid-derived 2)-like 2
KEAP1	Kelch-like ECH-associated protein 1
p62/SQSTM1	Sequestosome-1
ROS	Reactive oxygen species
GSH	Glutathione
GSR	Glutathione reductase
NQO1	NAD(P)H quinone dehydrogenase 1
HMOX1	Heme oxygenase 1
GPX1/2	Glutathione peroxidase 1/2
GST	Glutathione S-transferase
GCLC	Glutamate–cystein ligase catalytic subunit
GCLM	Glutamate–cystein ligase modifier subunit
SLC3A2	4F2 cell-surface antigen heavy chain
SLC7A11	Cystine/glutamate transporter
YAP1	Yes associated protein 1
NF-κB	Nuclear factor kappa-light-chain-enhancer of activated B cells
SCC	Squamous cell carcinoma
CHUK/IKKα	IκB kinase
