# Supplementary material for: Various Mechanisms Involve the Nuclear Factor (Erythroid-Derived 2)-Like (NRF2) to Achieve Cytoprotection in Long-Term Cisplatin-Treated Urothelial Carcinoma Cell Lines
Source: Int J Mol Sci. 2017 Aug 2;18(8):1680. doi: 10.3390/ijms18081680 (PMC5578070; doi:10.3390/ijms18081680)
Supplement: Supplementary file 1 [file ijms-18-01680-s001.pdf]

International Journal of *Molecular Sciences*

MDPI

Article

Various Mechanisms Involve the Nuclear Factor (Erythroid-Derived 2)-Like (NRF2) to Achieve Cytoprotection in Long-Term Cisplatin-Treated Urothelial Carcinoma Cell Lines

Margaretha A. Skowron [1], Günter Niegisch [1], Philipp Albrecht [2], Gommert van Koeveringe [3], Andrea Romano [4], Peter Albers [1], Wolfgang A. Schulz [1] and Michèle J. Hoffmann [1,*]

[1] Department of Urology, Medical Faculty, Heinrich-Heine-University, Duesseldorf 40225, Germany; Margaretha.Skowron@hhu.de (M.A.S.); Guenter.Niegisch@hhu.de (G.N.); peter.albers@med.uni-duesseldorf.de (P.A.); Wolfgang.Schulz@hhu.de (W.A.S.)

[2] Department of Neurology, Medical Faculty, Heinrich-Heine-University, Duesseldorf 40225, Germany; phil.albrecht@gmail.com

[3] Department of Urology, Maastricht University Medical Centre, Maastricht 6202AZ, The Netherlands; g.van.koeveringe@mumc.nl

[4] Department of Obstetrics and Gynaecology, GROW-School for Oncology & Developmental Biology, Maastricht University Medical Centre, Maastricht 6229HX, The Netherlands; a.romano@maastrichtuniversity.nl

* Correspondence: Michele.Hoffmann@hhu.de; Tel.: +49-211-8115847

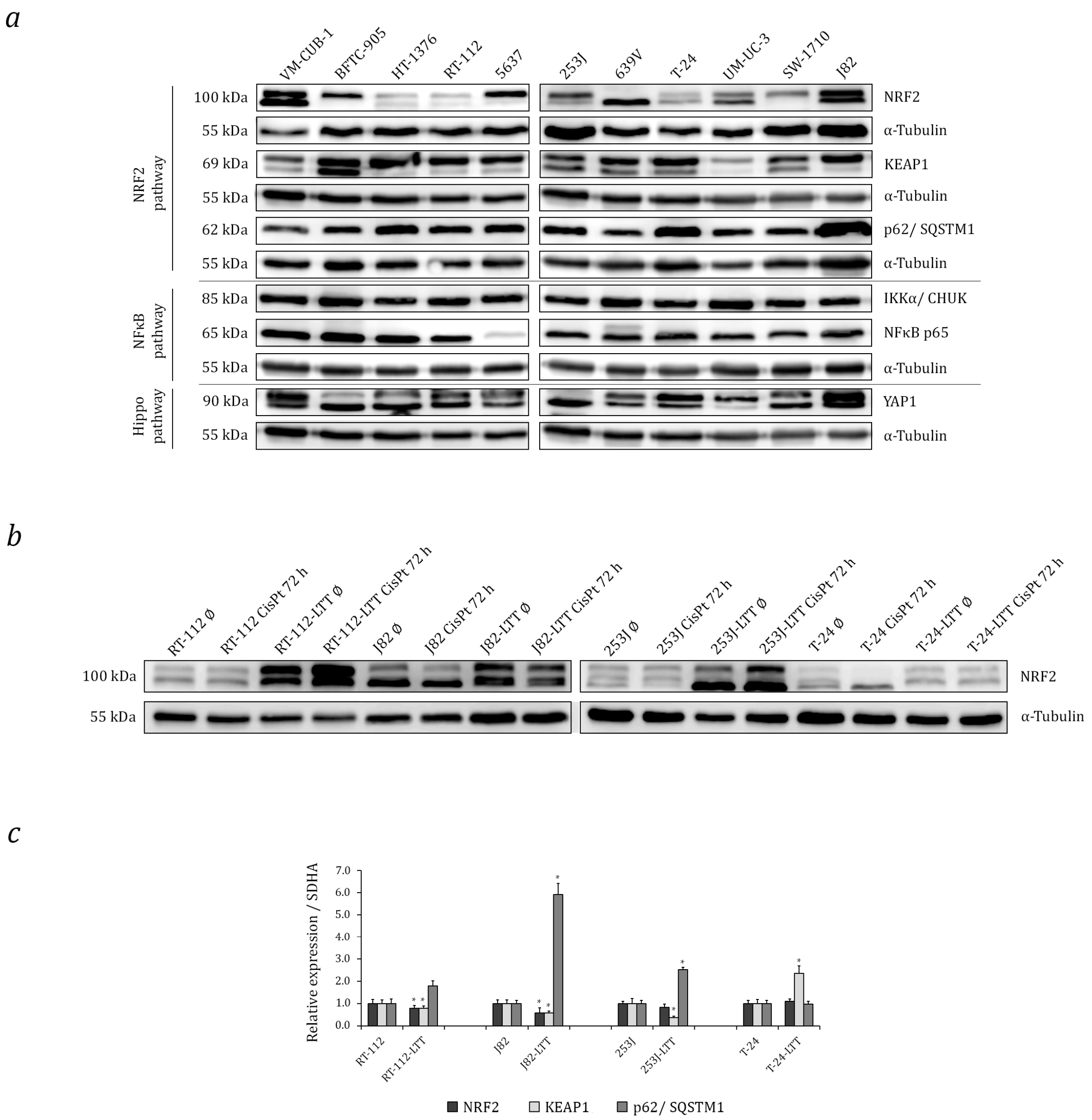

Figure S1. Stable nuclear factor (erythroid-derived 2)-like 2 (NRF2) upregulation in long-term cisplatin treated cell lines (LTTs). (***a***) Yes associated protein 1 (YAP1), Kelch-like ECH-associated protein 1 (KEAP1), NRF2, sequestosome-1 (p62/SQSTM1), IκB kinase (IKK*α*), and nuclear factor kappa-light-chain-enhancer of activated B cells (NF-κB p65) protein expression were measured among 11 urothelial carcinoma cell lines (UCCs). (***b***) NRF2 protein expression was measured in four untreated and 72 h cisplatin treated parental UCCs and their long-term cisplatin treated cell lines (LTTs) 72 h and 10 days after cisplatin treatment. As a loading control, *α*-Tubulin was detected. (***c***) NRF2, KEAP1, and p62/SQSTM1 mRNA expression in LTTs and their parental cell lines was measured by qRT-PCR. Expression levels in the untreated control were set as 1. SDHA mRNA was used as a reference and relative expression was calculated by the $2^{-\Delta\Delta Ct}$ method. Values represent the mean ± SD of biological triplicates. * $p < 0.05$.

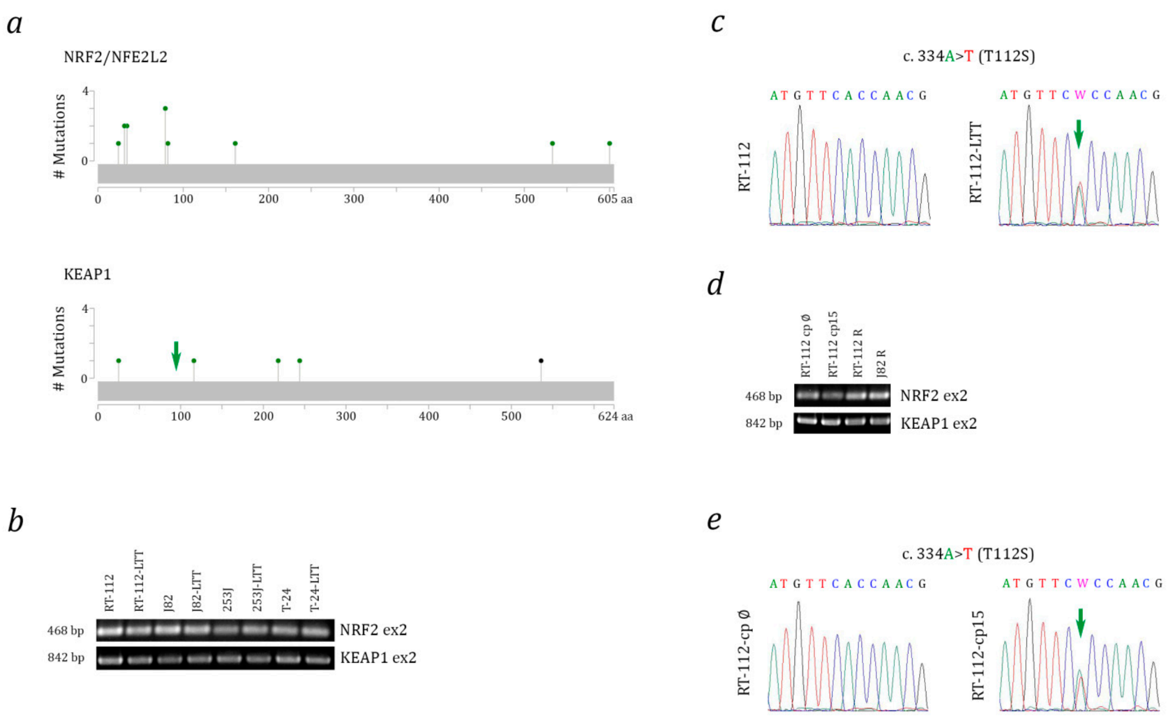

Figure S2. *NFEL2L* or *KEAP1* mutation as a potential factor in cisplatin resistance. (***a***) Annotated *NFEL2L* and *KEAP1* mutations according to data obtained by the cBioportal data base [46,47]. Green and black lollipops indicate missense and truncating mutations, respectively. Green arrow indicates mutation found in RT-112-LTT as shown in (***c***). (***b***) Qualitative PCR of *NFEL2L* exon 2 and *KEAP1* exon 2 in four LTTs and their parental cell lines. (***c***) Mutation found in *KEAP1* exon 2 in RT-112-LTT compared to unmodified parental RT-112 by Sanger sequencing. (***d***) Qualitative PCR of *NFEL2L* exon 2 and *KEAP1* exon 2 in RT-112 and J82 from different cisplatin selection protocols (RT-112cp15 [51], RT-112-R, and J82-R [45]). (***e***) Mutation found in *KEAP1* exon 2 in RT-112cp15 compared to unmodified parental RT-112cp [51] by Sanger sequencing.

Table S1. Summary of qRT-PCR data. Up (↑), downregulated (↓), and unchanged (−) mRNA levels of several genes involved in the NRF2 and orchestrating pathways, cytoprotective enzymes, and glutathione (GSH) biosynthesis. n.d.: not detectable, * $p < 0.05$, ** $p < 0.01$.

	**Factor**	**Function**	**RT-112 / RT-112-LTT**	**J82 / J82-LTT**	**253J / 253J-LTT**	**T-24 / T-24-LTT**
NRF2 pathway	NRF2	NRF2 pathway	↓*	↓*	−	−
	KEAP1	NRF2 pathway	↓**	↓*	↓*	↑**
	p62/SQSTM1	NRF2 pathway	−	↑**	↑**	−
	CHUK	NF-κB pathway	−	↓**	↓**	−
	RELA	NF-κB pathway	−	↓**	↓**	−
	YAP1	Hippo pathway	−	−	−	↓*
Cytoprotective enzymes	GSR	Inactivation	↑**	−	−	↑*
	NQO1	Inactivation	↑**	−	−	↑*
	GPX1	Inactivation	↑**	−	−	↑**
	GPX2	Inactivation	↑*	*n.d.*	−	↑*
	GSTM1	Inactivation	↑**	↓**	−	↓**
	GSTP1	Inactivation	↑**	−	−	↑**
GSH biosynthesis	GCLM	GSH synthesis	↑**	↓**	−	↑**
	GCLC	GSH synthesis	↓**	↓**	−	↑*
	SLC3A2	GSH transport	↑**	↑**	↓**	↑**
	SLC7A11	GSH transport	↑**	↓**	−	↑**

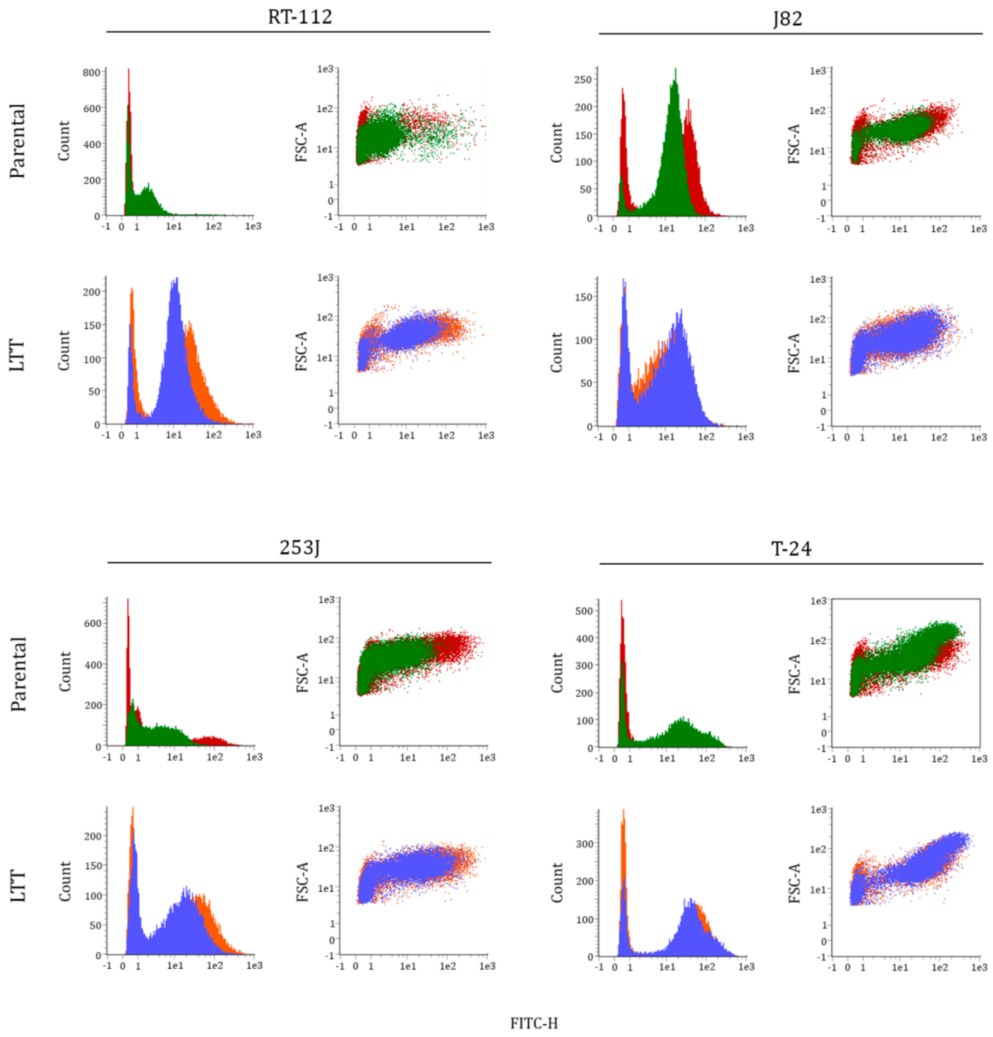

Figure S3. Decreased accumulation of reactive oxygen species (ROS) in most LTTs. Intracellular ROS accumulation was analysed by 2′,7′-Dichlorodihydrofluorescein Diacetate (DCFH-DA) staining and was measured in parental UCCs (green) and LTTs (blue) after 72 h cisplatin treatment (red and orange, respectively) by flow cytometry. Values represent the mean ± SD of biological quadruplicates.

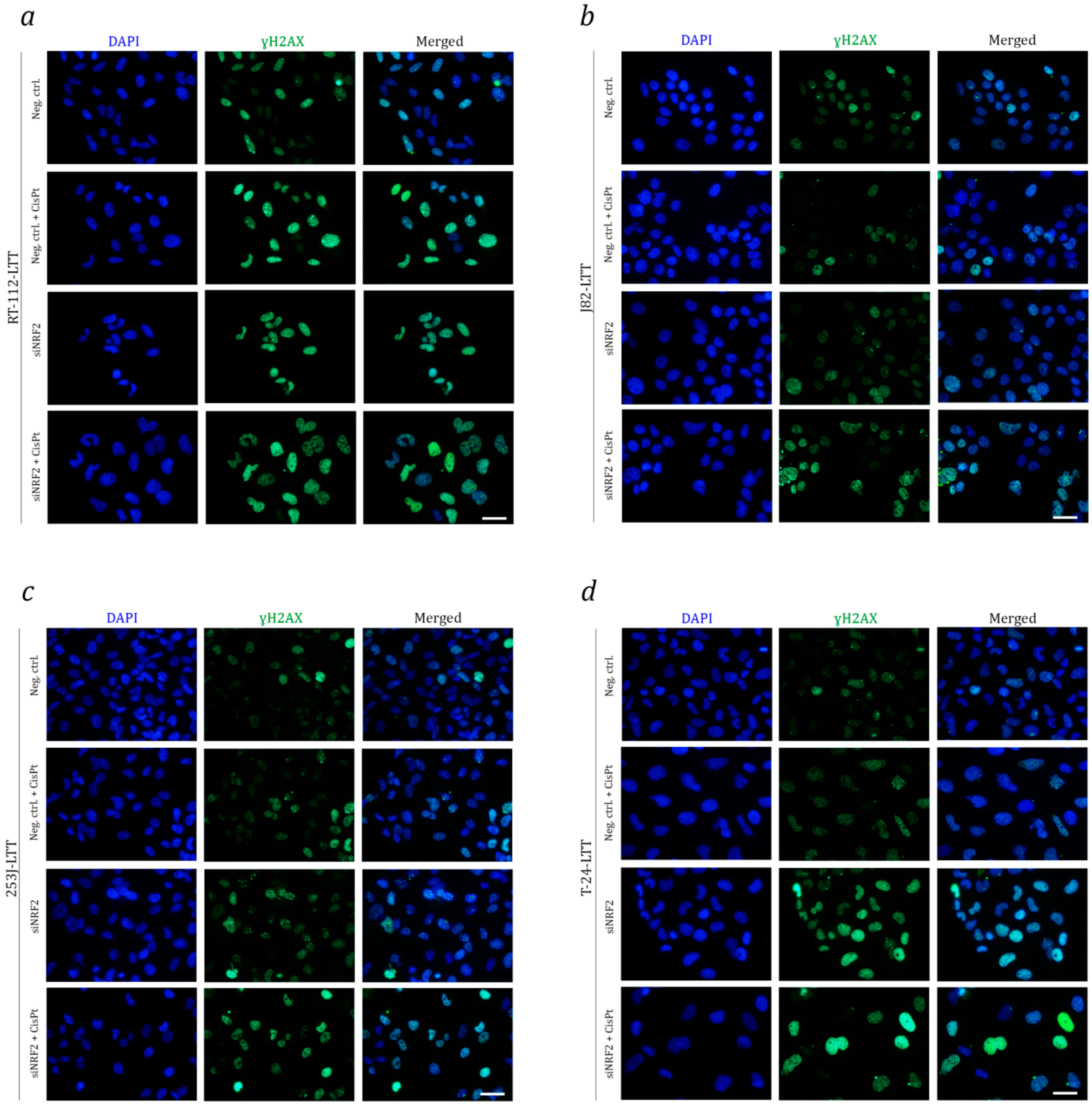

Figure S4. NRF2 knockdown sensitises LTTs towards cisplatin by increasing DNA damage. Representative immunofluorescence staining for pH2A.X Ser139 foci in siNRF2- or control siRNA-transfected LTTs after 72 h cisplatin treatment in (***a***) RT-112-LTT, (***b***) J82-LTT, (***c***) 253J-LTT, and (***d***) T-24-LTT compared to their untreated controls.

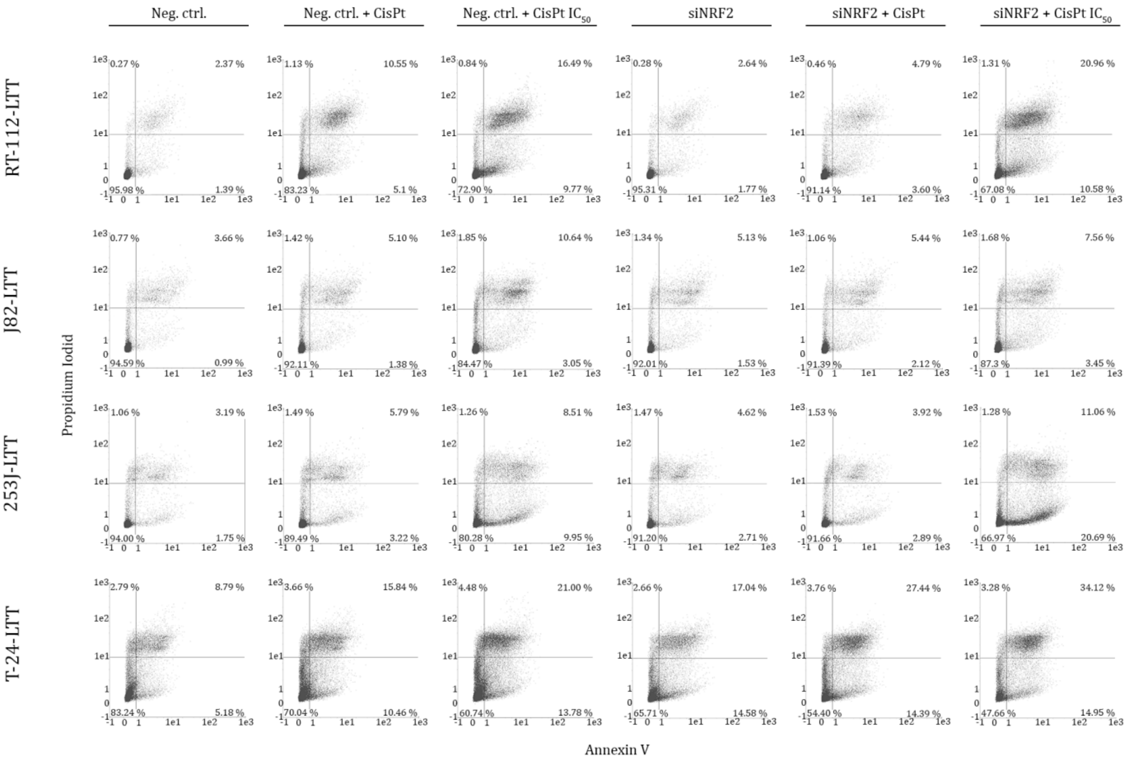

Figure S5. NRF2 knockdown sensitises LTTs towards cisplatin by induction of cell death. Induction of necrosis and apoptosis was analysed in siNRF2- or control siRNA-transfected LTTs 72 h after cisplatin treatment (maintenance and half maximal inhibitory concentration (IC_{50})) by combined Annexin V and PI staining with subsequent flow cytometry. The percentages in the figure indicate viable cells (bottom left), necrotic cells (top left), early apoptotic cells (bottom right), and late apoptotic/necrotic cells (top right).

Table S2. Primer sequences for quantitative real-time-PCR. Sequences of primers (5′-3′) used for quantitative real-time-PCR including length of PCR products, accession numbers, and annealing temperatures. bp.: base pair; Fwd.: Forward; Rev.: Reverse.

Gene Name	Accession Number	Size [bp]	Sequence 5′-3′	$T_{Annealing}$ [°C]
SDHA	NM_004168	140	Fwd.: GCCAGGACCTAGAGTTTGTTCA<br>Rev.: CTTTCGCCTTGACTGTTAATGA	55
IKKα/CHUK	NM_001278	115	Fwd.: TGCCTTGGCCATTTAAGCACTA<br>Rev.: GGGACAGTGAACAAGTGACAACTC	57
GCLC	NM_001197115	105	Fwd.: GATGCTGTCTTGCAGGGAATG<br>Rev.: AGCGAGCTCCGTGCTGTT	58
GCLM	NM_002061	239	Fwd.: TGTCTTGGAATGCACTGTATCTC<br>Rev.: CCCAGTAAGGCTGTAAATGCTC	55
GPX1	NM_201397	104	Fwd.: ACGATGTTGCCTGGAACTTT<br>Rev.: GATGTCAGGCTCGATGTCAA	53
GPX2	NM_002083	174	Fwd.: GGTAGATTTCAATACGTTCCGGG<br>Rev.: TGACAGTTCTCCTGATGTCCAAA	52
GSR	NM_001195102	115	Fwd.: ACAAGCTGGGTGGCACTT<br>Rev.: CAACTTGGAAAGCCATAA	51
GSTM1	NM_000561	87	Fwd.: ACTATCCTTCGTGAACATC<br>Rev.: AGACACAACCACTAACAG	50
GSTP1	NM_000852	148	Fwd.: ACCTCCGCTGCAAATACATC<br>Rev.: TGGTCTCCCACAATGAAGGT	54
HMOX1	NM_002133	220	Fwd.: CTCAAACCTCCAAAAGCC<br>Rev.: TCAAAAACCACCCCAACCC	55
KEAP1	NM_012289	64	Fwd.: GTGTGGAGAGGTATGAGCCA<br>Rev.: CTTCGTGTCAGCATTGGG	54
NF-κB p65/RELA	NM_001243985	112	Fwd.: TCTGCTTCCAGGTGACAGTG<br>Rev.: ATCTTGAGCTCGGCAGTGTT	55
NRF2/NFE2L2	NM_006164	83	Fwd.: ACACGGTCCACAGCTCATC<br>Rev.: TGTCAATCAAATCCATGTCCTG	54
NQO1	NM_001025434	121	Fwd.: TCACCGAGAGCCTAGTTCC<br>Rev.: CTGAGTGAGCCAGTACGATC	56
P62/SQSTM1	NM_003900	137	Fwd.: GTGGTAGGAACCCGCTACAA<br>Rev.: GAGAAGCCCTCAGACAGGTG	57
SLC3A2	NM_002394	201	Fwd.: ACGGTGGTGTGTTTGCTGTCT<br>Rev.: AGGAGTGTGCTTGCGGACAT	55
SLC7A11	NM_014331	101	Fwd.: CTGGTGCCGTGGTCATAATC<br>Rev.: GAATTGGACCCAGCACAAGG	56
YAP1	NM_006106	131	Fwd.: CGCTCTTCAACGCCGTCA<br>Rev.: AGTACTGGCCTGTCGGGAGT	56

Table S3. Primer sequences for qualitative PCR. Sequences of primers (5′-3′) used for qualitative PCR including length of PCR products and annealing temperatures. bp.: base pair; Fwd.: Forward; Rev.: Reverse.

Gene Name	Exon	Accession Number	Size [bp]	Sequence 5′-3′	T Annealing [°C]
NRF2/NFE2L2	2	NM_006164	468	Fwd.: CCACTTCCCACCATCAACAG<br>Rev.: GAAAGGCAAAGCTGGAACTCA	55
KEAP1	2	NM_012289	842	Fwd.: TATCTTGCAAAACGAGGCCC<br>Rev.: AAGGGGAGACAGTGATGAGC	58
